# The Influence of Visual Input on Electromyographic Activity and Patterns of Masticatory and Cervical Spine Muscles in Emmetropic Caucasian Subjects by Gender

**DOI:** 10.3390/ijerph20054112

**Published:** 2023-02-25

**Authors:** Grzegorz Zieliński, Anna Matysik-Woźniak, Maria Rapa, Michał Baszczowski, Beata Pająk, Michał Ginszt, Jacek Szkutnik, Robert Rejdak, Piotr Gawda

**Affiliations:** 1 Department of Sports Medicine, Medical University of Lublin, 20-093 Lublin, Poland; 2 Department of General and Pediatric Ophthalmology, Medical University of Lublin, 20-093 Lublin, Poland; 3 Students’ Scientific Association at the Department and Clinic of General and Pediatric Ophthalmology, Medical University of Lublin, 20-093 Lublin, Poland; 4 Interdisciplinary Scientific Group of Sports Medicine, Department of Sports Medicine, Medical University of Lublin, 20-093 Lublin, Poland; 5 Department of Rehabilitation and Physiotherapy, Medical University of Lublin, 20-093 Lublin, Poland; 6 Independent Unit of Functional Masticatory Disorders, Medical University of Lublin, 20-093 Lublin, Poland

**Keywords:** emmetropic, activity index, asymmetry index, electromyography, masticatory muscles, temporomandibular joint, myopia, hyperopia, vision, optometry

## Abstract

(1) Background: The objective of the research was to analyze the change of visual input on electromyographic activity and patterns of masticatory and cervical spine muscles in emmetropic Caucasian subjects by gender. Supposedly, visual input should not influence activity and electromyographic patterns of masticatory and cervical spine muscles in emmetropic Caucasian subjects by gender. (2) Methods: After applying the inclusion criteria, 50 emmetropic Caucasian subjects were included in the study. Four muscle pairs were analyzed: the temporalis muscle (TA), the masseter muscle (MM), the digastric muscle (DA), and the sternocleidomastoid muscle (SCM), during resting and functional activity. (3) Results: It was observed that there were no significant statistical differences in activity and bioelectrical patterns between open and closed eyes in women and men, with the exception of clenching on dental cotton rollers in DA-left and DA mean between tests in women. The observed statistical results had a small effect size, successively equal to 0.32 and 0.29. (4) Conclusions: Changes in the influence of visual input do not affect electromyographic activity and patterns of masticatory and cervical spine muscles in emmetropic Caucasian women and men.

## 1. Introduction

The eye is an anatomically complex organ, which decides to a significant extent our perception of reality. The ability to see is the result of a complex process, in which the eye receives light from the environment and then converts it into energy. Such energy produces action potential in dedicated nerve cells. Next, it is transmitted through the second cranial nerve (optic nerve) to the brain, where it is interpreted [1,2]. Visual acuity is related to the normal refraction of the light that passes through the structures of the eyeball, including the cornea, aqueous humor, lens, and vitreous humor before reaching the retina. The retina contains light-sensitive receptor cells: rods and cons [1].

In the simplest terms, refractive error is a problem with focusing the light accurately on the retina, due to the shape of the eye. The most common types of refractive errors are: myopia, hyperopia, astigmatism, and presbyopia [3]. Studies show connections between refractive errors (myopia) and changes in the stomatognathic system.

This system is a functional complex of tissues and organs located in the craniofacial and oral cavities [4]. Studies link blurred vision (in the absence of correction) to higher muscle bioelectric activity in the stomatognathic system compared to muscle activity with eyes closed [5,6]. It is not possible to say how exactly it is linked, but the vestibulo-ocular reflex (VOR) [4], central sensitization, and changes in the musculo-fascial network [4,5,6,7] are considered here [4].

Connections between the musculo-fascial system and the sight organ are also observed in people without a refractive error. A correlation has been observed between the axial length of the eyeball, bioelectrical activity [8], and the thickness of selected muscles of the masticatory organ [7]. Such connections have also been found between retinal thickness and intraocular pressure in subjects without any defect and the activity as well as thickness of the muscles [7].

Since myopia is one of the most common refractive errors causing eye disease in children and young adults in particular [9], research on the connection between the stomatognathic system and the organ of vision has focused more on ill patients than on healthy individuals. Based on the literature, the authors accessed a few studies addressing the effect of visual stimulus as determined by the closed versus open eye test in emmetropic subjects. 

A study performed by Widmalm and Ericsson (1983) (n = 4) shows that closing both eyes reduces muscle activity by 50–100% [10]. Miralles et al. (1998) (n = 18) observed a decrease in bioelectrical activity at rest, but they did not notice any change in clenching in the intercuspal position in the aforementioned test [11]. Monaco et al. (2006) (n = 10) [12], Spadaro et al. (2010) (n = 20) [13], and Ciavarella et al. (2014) (n = 15) [14] showed no differences in bioelectrical activity of selected muscles in the test.

The above studies do not present conclusive results. They are conducted on less than 20 subjects, and they do not analyze changes within the activity indexes (AcI), and asymmetry indexes (AsI) (in which significant changes came out in subjects with myopia [6]). The above papers also do not differentiate subjects by gender, which can be an important factor [15]. Additionally, they do not analyze whether subjects had measured intraocular pressure or eyeball length (the aforementioned variables are associated with changes in surface electromyography (sEMG) signal [7,8]). What is more, the abovementioned studies do not indicate the subjects’ race. Due to these factors, the authors of this paper decided to conduct the present study.

The objective of the research was to analyze the change of visual input on electromyographic activity and patterns of masticatory and cervical spine muscles in emmetropic Caucasian subjects by gender. Based on the lack of sufficient evidence and methodological shortcomings in the works presented above, the following research hypothesis has been put forward: supposedly, the visual input should not influence activity and electromyographic patterns of masticatory and cervical spine muscles. To the best of our knowledge, this is the first study that analyzes changes in electromyographic activity and patterns of masticatory and cervical spine muscles in emmetropic Caucasian subjects by gender.

## 2. Materials and Methods

A total of 103 subjects were enrolled in the study. The study lasted from April 2021 to December 2021. The following inclusion criteria were used:

- no refractive error;
- no ocular and optic nerve diseases;
- an eye with a measured axial length of more than 23 mm [16] and less than 24 mm [17] based on the IOL Master 500 test (Carl Zeiss Meditec, Jena, Germany) [7];
- absence of temporomandibular disorders according to the two-axis Research Diagnostic Criteria for Temporomandibular Disorders (RDC/TMD) [18];
- absence of musculoskeletal and connective tissue diseases and absence of myofascial trigger points as determined by the gold standard diagnostic criteria set by Travell and Simons [19] (within the temporalis muscle (TA), the masseter muscle (MM), the sternocleidomastoid muscle (SCM), the digastric muscle (DA), the upper part of the trapezius muscle),
- absence of malignancy (regardless of type and location);
- absence of metal implants (regardless of type and location).
- The exclusion criteria:
- intraocular pressure above 20 mmHg as determined by the Tono-Pen XL (Medtronic Solan, FL, USA) [7];
- no visual acuity of 1.0 as assessed by the Snellen chart on monocular examination [20];
- any malocclusion;
- oral inflammation;
- orthodontic treatment;
- race other than Caucasian;
- trauma and surgical treatment of the head and neck within the last 6 months.

The tests for inclusion and exclusion criteria were conducted by ophthalmologist (A.M-W.), dental prosthetics specialist (J.S.) and physiotherapist (G.Z.).

The study was approved by the local Ethics Committee of the Medical University of Lublin (approval number KE-0254/229/2020). Written consent was obtained from all respondents who participated in the study.

After applying the inclusion criteria, 50 subjects were included in the study. The characteristics of the group are shown in Table 1.

The surface electromyography (sEMG) study was performed using an 8-channel BioEMG III electromyograph (BioResearch Associates, Inc., Milwaukee, WI, USA). Four muscle pairs were analyzed: the anterior part of the TA; the superficial part of the MM; the middle part of the SCM; the anterior belly of the DA. The subjects assumed standardized positions in the dental chair—consistent with the previous methodology [5,6,7,8,21]. After cleansing the skin with ethyl alcohol, disposable Ag/AgCl electrodes (SORIMEX, Toruń, Poland) were applied, and a reference electrode was placed on the forehead. The sEMG test followed surface EMG for a non-invasive assessment of muscles (SENIAM) protocol [22].

Electromyographic activity was recorded in four activities:

- at rest (10 s);
- during maximal voluntary clenching in the intercuspal position (as hard as possible; 3 × 3 s, 2 s rest between);
- during maximal voluntary clenching on dental rollers (as hard as possible; 3 × 3 s, 2 s rest between);
- during maximal mouth opening (as wide as possible; 3 × 3 s, 2 s rest between) [5,6,8,23,24,25].

The sEMG test was conducted with eyes open and eyes closed with a 5 min break between tests. There was a random selection of the initial test. A random number generator was used for this where 1 meant an open eye test and 2 meant a closed eye test. [5,6,8,12,13,14].

**Figure 1:**
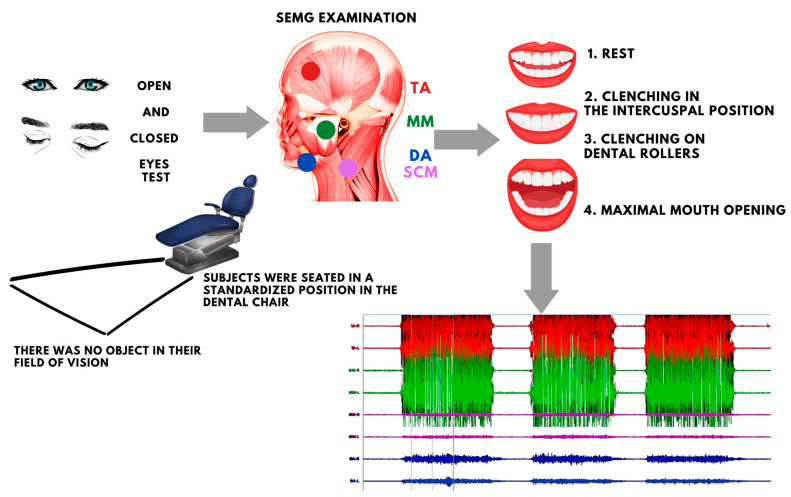
Schematic order of the study. TA—the anterior part of the temporalis muscle; MM—the superficial part of the masseter muscle; SCM—the middle part of the sternocleidomastoid muscle; DA—the anterior belly of the digastric muscle.

Software compatible with the sEMG processed the electromyographic signal automatically, based on root means square (RMS) in the BioPAK program. It allowed us to obtain an average measurement of values, which were then used to analyze muscle activity (Figure 1). The study was conducted by the same researcher, who specializes in electromyography (G.Z.).

Based on the RMS, the activity and asymmetry indices (AcI and AsI) proposed by Naeije et al. [26] were counted.

(1) $$\mathrm{Activity}\;\mathrm{Index}\;\left(\mathrm{AcI}\right)=\frac{RMS_{MM}-RMS_{TA}}{RMS_{MM}+RMS_{TA}}\times 100$$

AcI values range between +100% and −100%, with additional values determining the proportion of masseter muscle activity over time and negative values for the temporalis muscle [26].

(2) $$\mathrm{Asymmetry}\;\mathrm{Index}\;\left(\mathrm{AsI}\right)=\frac{RMS_{\mathrm{right}}-RMS_{\mathrm{left}}}{RMS_{\mathrm{right}}+RMS_{\mathrm{left}}}\times 100$$

AsI values range between +100% and −100%, with additional values determining the proportion of muscles tested on the right side, and negative values on the left side. In both cases, a value of 0 defines perfect symmetry [26].

The data comparison was performed using the GraphPad Prism 9.4.1 program (GraphPad Software, Inc., San Diego, CA, USA). The normality of the distribution of variables was verified using the Shapiro–Wilk test and the Kolmogorov–Smirnov test (with the Lillierfors correction). The values did not have a normal distribution; therefore, the Mann–Whitney U test (Z) was used to compare the differences between groups. Effect sizes were determined for the t-test using the Cohen d method and interpreted as small (0.2), medium (0.5), and large (0.8) effect sizes [27,28]. A confidence interval (CI 95%) was calculated for results at the level of 95% [29]. The calculations indicated that a sample size of 17 participants would be sufficient to notice significant differences between matched pairs (*t*-test) with an α value of 0.05, a power value of 0.90, and an estimated medium effect size of 0.75. The sample size analysis was carried out using the G*Power 3.1 program (Heinrich-Heine-Universität Düsseldorf, Düsseldorf, Germany) [30]. Statistical significance was set at *p* ≤ 0.05. The results were presented graphically using the GraphPad Prism 9.4.1 program (Dotmatics, Boston, MA, USA).

## 3. Results

**Figure 2:**
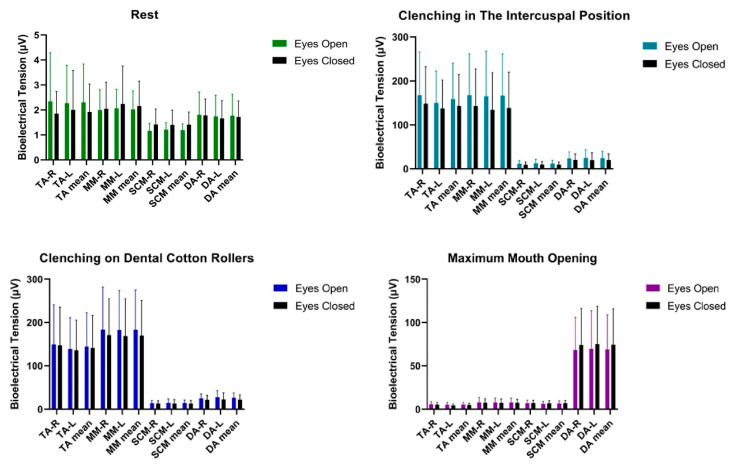
Graphical representation of bioelectrical activity of selected muscles of the masticatory and cervical spine muscles in women. TA—the anterior part of the temporalis muscle; MM—the superficial part of the masseter muscle; SCM—the middle part of the sternocleidomastoid muscle; DA—the anterior belly of the digastric muscle; R—right site; L—left site.

Statistical analysis of bioelectrical activity conducted on the group of women did not show any significant statistical differences in the tested muscle activities of TA, MM, SCM, DA except for clenching on dental cotton rollers in DA-L and DA mean between tests. The observed statistical results had a small effect size, successively equal to 0.32 and 0.29. The confidence interval was at the level for DA-L -−9.40, −0.80 for DA mean −9.05, −0.25 Table 2 and Figure 2.

**Figure 3:**
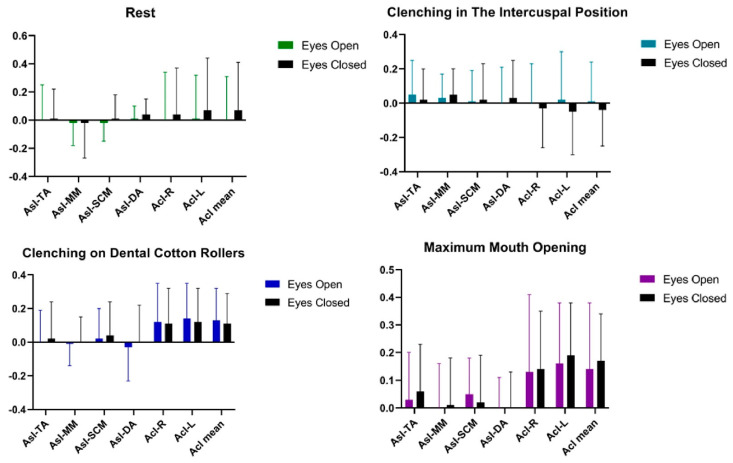
Graphical representation of bioelectrical patterns of selected muscles of the masticatory and cervical spine muscles in women. AcI—Activity index; AsI—Asymmetry Index; TA—the anterior part of the temporalis muscle; MM—the superficial part of the masseter muscle; SCM—the middle part of the sternocleidomastoid muscle; DA—the anterior belly of the digastric muscle.

Statistical analysis of AcI and AsI patterns conducted on the women’s group showed no significant statistical differences in the tested muscle activities of TA, MM, SCM, DA between the tests Table 3 and Figure 3.

**Figure 4:**
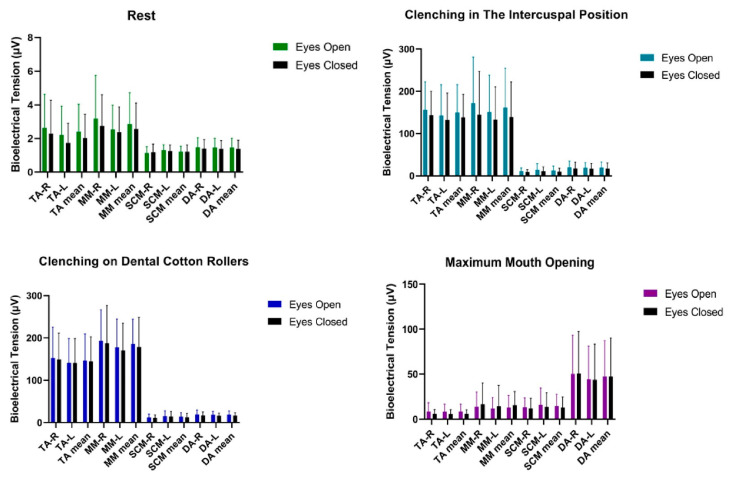
Graphical representation of bioelectrical activity of selected muscles of the masticatory and cervical spine muscles in men. TA—the anterior part of the temporalis muscle; MM—the superficial part of the masseter muscle; SCM—the middle part of the sternocleidomastoid muscle; DA—the anterior belly of the digastric muscle; R—right site; L—left site.

Statistical analysis of bioelectrical activity performed on the male group showed no significant statistical differences in the tested muscle activities of TA, MM, SCM, DA between the tests (Table 4 and Figure 4).

**Figure 5:**
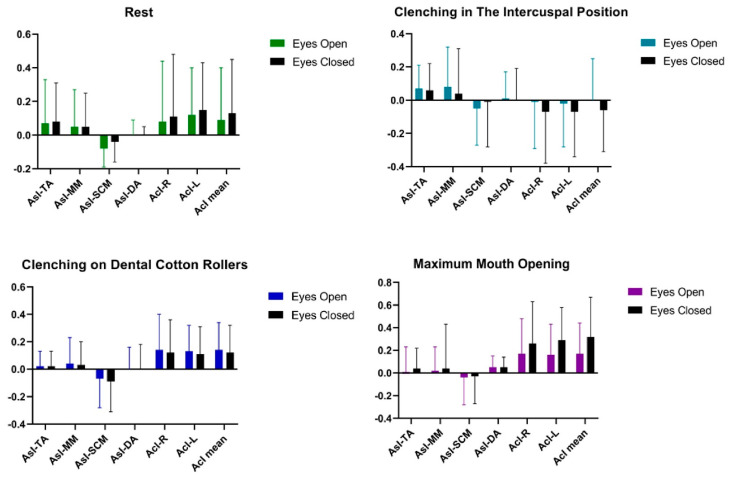
Graphical representation of bioelectrical patterns of selected muscles of the masticatory and cervical spine muscles in men. AcI—Activity index; AsI—Asymmetry Index; TA—the anterior part of the temporalis muscle; MM—the superficial part of the masseter muscle; SCM—the middle part of the sternocleidomastoid muscle; DA—the anterior belly of the digastric muscle.

Statistical analysis of AcI and AsI patterns conducted on a group of men showed that there were no significant statistical differences in the tested muscle activities of TA, MM, SCM, DA between the tests (Table 5 and Figure 5).

## 4. Discussion

This study aims to analyze the change of visual input on electromyographic activity and patterns of masticatory and cervical spine muscles in emmetropic Caucasian subjects by gender. The purpose of the study was accomplished, and the research hypothesis was confirmed. Emmetropic Caucasian women and men show no changes in the bioelectrical activity of muscles and no changes in TA, MM, SCM, and DA bioelectrical patterns. The observed statistical differences in clenching on dental cotton rollers in DA-L and DA mean between the tests in women show a small effect size (effect size in sequence 0.32 and 0.29 [27,28]). It is only seen with cotton rollers which do not represent functional activity. Our observations are consistent with the findings of Monaco et al. [12], Spadaro et al. [13], and Ciavarella et al. [14]. 

No statistical differences in most analyses can be related to the lack of excitation of the trigeminal nuclei in the VOR reflex pathway. This reflex belongs to the group of unconditioned reflexes. It allows correct alignment of the optical axes of the eyes and binocular vision [31]. According to Flourens and Ewald’s law, it is known that the plane of rotation of the nystagmus is in line with the plane of the excited semicircular canal [32]. By the VOR, the medial vestibular nucleus sends excitatory signals to the abducent nucleus of the contralateral side, and at the same time inhibitory signals to the abducent nucleus of the ipsilateral side [31]. The sensory nuclei of the trigeminal nerve are so large that they mediate the impulses of all cranial nerves [4,33]. Hypothetically, at this stage of nerve impulse transmission through the VOR reflex pathway, the trigeminal nuclei are stimulated and send an impulse to the muscles of the masticatory organ, which causes an increase in the activity of the muscles of the masticatory organ, which further stabilizes the craniofacial region (in people with uncorrected myopia, stimulation of the trigeminal nuclei is increased) [4]. This brings to the foreground the explanation for the changes in myopic subjects during the open-eye versus closed-eye test [5,7,12,14,21]. It is worth noting the statistically insignificant decrease in bioelectrical activity with eyes closed (Table 2 and Figure 2; Table 4 and Figure 4), which confirms the involvement of trigeminal nuclei in cranial nerve impulses (abducens nerve, vestibulocochlear nerve and accessory nerve) [4,33]. Hypothetically in the emmetropic subjects, a modification in visual stimulus does not trigger a significant potential that might occur in changes.

Another explanation is the lack of musculo-fascial changes in the cranial and neck muscles in the emmetropic subjects. The connection along this way occurs from the Tenon’s capsule that surrounds the wall of the eyeball from the edge of the ciliary body to the entrance of the optic nerve in an inward direction. Externally, Tenon’s capsule connects to the deep fascia of the cranium (cranial fascia) and through it to the temporal fascia [34]. According to a new study (2022), during analysis of the correlation between bioelectric tension and eye length in the myopic subjects and emmetropic subjects, the positive correlation was observed. However, the emmetropic subjects had more correlations than the myopic person [8]. Hypothetically, in subjects without refractive error, fascial sliding is not disrupted by fascial densifications caused, for example, by a reduction in the visual field through the wearing of glasses or a significant elongation of the eyeball (as occurs in axial myopia). This may explain the lack of observed changes. Future studies should examine how the weight of glasses affects changes in the muscular system and how the system adapts to years of wearing glasses.

Our results confirm that changing the visual stimulus from the open eyes test to closed eyes test, does not cause changes in bioelectrical activity in emmetropic Caucasian women and men. The lack of change in emmetropic subjects vs. significant change in bioelectrical activity and electromyographic patterns in subjects with myopia (proven in other studies [5,6,12,21], higher bioelectrical activity with open eyes [5,12]) suggests that sEMG can be a form of test to control the correctness of the selection of vision correction (glasses, lenses). It needs further research. It can also be a useful diagnostic tool in tension-type headaches (to analyze the possible contribution of visual factors) and can improve the diagnosis of visual defects in unaware patients. The above diagnostic possibilities are also made for further research perspectives recommended by the authors. The reason that the refractive defect is race-dependent [35,36] is also worth checking with the replicability of the results obtained on other races.

The study presented here has several limitations. First, the study was conducted on a Caucasian population. We suggest checking the results obtained on other races. The study was conducted on young adults, and we suggest analysis in other age ranges. The effects of occlusion and mandibular position on muscle and posture are debatable topics. Some research links mandibular and occlusion positions to postural changes [37,38]. However, previous studies have not observed an effect of occlusion on changes in masticatory muscle activity [23,24,39]. Therefore, we suggest in future studies to analyze other human muscles. A final limitation is that the diagnostic criteria for TMD changed to The Diagnostic Criteria for Temporomandibular Disorders (DC/TMDs) in 2014. However, the present study used the previous version. To date, there is no validated Polish version of DC/TMDs, so RDC/TMDs was used.

## 5. Conclusions

Changes in the influence of visual input do not affect the electromyographic activity and electromyographic patterns of masticatory muscles and cervical spine in the emmetropic Caucasian population. We suggest expanding the study to other races, a different age range and other muscles of the body.

## Figures and Tables

**Table 1:** Characteristics of the group—summary.

		Women		Men	
		Mean	SD	Mean	SD
n		32.00		18.00	
Age (years)		23.09	1.89	23.67	2.45
Height (cm)		167.38	7.17	179.72	4.74
Weight (kg)		59.90	8.72	78.50	6.82
Body Mass Index		21.38	2.96	24.31	1.93
Visual Acuity	R	1.0		1.0	
	L	1.0		1.0	
Intraocular Pressure (mmHg)	R	15.61	3.70	14.50	3.17
	L	14.44	3.90	14.40	3.81
Axial Length (mm)	R	23.48	0.53	23.95	0.63
	L	23.46	0.52	23.95	0.60
Mandibular Range Of Motion (mm)	Abduction Without Pain	49.44	4.66	53.28	6.73
	Active Abduction	50.69	5.71	54.39	6.18
	Passive Abduction	52.97	5.88	57.17	6.21
	Mandibular Movement to The Right	9.16	2.85	9.89	2.93
	Mandibular Movement to The Left	10.03	2.72	9.67	2.54
	Protrusion	8.72	2.76	8.56	1.98

R—right site; L—left site.

**Table 2:** Results of comparison of bioelectrical activity of selected muscles of the masticatory and cervical spine muscles in women.

		Open Eye		Closed Eye					
		Mean	SD	Mean	SD	Z	P	CI 95%	
Rest	TA-R	2.34	1.95	1.85	0.89	0.43	0.67	−0.57	0.29
	TA-L	2.27	1.52	2.00	1.58	1.18	0.24	−0.74	0.22
	TA mean	2.30	1.54	1.92	1.12	0.73	0.47	−0.69	0.21
	MM-R	1.99	0.82	2.05	1.06	0.16	0.87	−0.45	0.42
	MM-L	2.06	0.77	2.24	1.52	0.21	0.83	−0.47	0.36
	MM mean	2.02	0.74	2.15	1.00	−0.03	0.98	−0.40	0.40
	SCM-R	1.16	0.31	1.42	0.62	−1.55	0.12	−0.03	0.38
	SCM-L	1.21	0.28	1.40	0.59	−0.85	0.39	−0.09	0.25
	SCM mean	1.19	0.25	1.41	0.51	−1.56	0.12	−0.04	0.33
	DA-R	1.80	0.92	1.78	0.66	−0.62	0.53	−0.21	0.43
	DA-L	1.74	0.85	1.66	0.71	0.11	0.91	−0.30	0.27
	DA mean	1.77	0.86	1.72	0.64	−0.19	0.85	−0.24	0.36
Clenching in The Intercuspal Position	TA-R	167.40	98.62	148.17	83.93	0.64	0.52	−56.60	28.60
	TA-L	149.59	72.71	137.48	64.42	0.68	0.50	−46.50	23.90
	TA mean	158.49	81.56	142.82	72.11	0.76	0.45	−50.50	26.80
	MM-R	167.43	94.37	142.62	84.51	1.23	0.22	−60.10	16.40
	MM-L	164.69	103.30	134.35	84.37	1.26	0.21	−63.90	15.10
	MM mean	166.06	95.45	138.48	81.51	1.26	0.21	−61.15	14.70
	SCM-R	11.60	6.96	9.45	6.26	1.56	0.12	−4.70	0.60
	SCM-L	12.26	9.61	9.73	7.06	1.26	0.21	−5.00	1.10
	SCM mean	11.93	7.72	9.59	5.99	1.28	0.20	−4.90	0.90
	DA-R	23.53	14.92	20.25	13.57	1.09	0.28	−8.00	2.50
	DA-L	24.65	18.70	19.84	16.79	1.52	0.13	−9.00	1.20
	DA mean	24.09	15.50	20.05	13.95	1.34	0.18	−9.05	1.55
Clenching on Dental Cotton Rollers	TA-R	149.47	91.32	147.11	88.71	−0.01	0.99	−45.10	41.40
	TA-L	139.13	72.32	135.68	69.44	0.11	0.91	−34.80	30.70
	TA mean	144.30	78.40	141.39	75.18	0.15	0.88	−41.00	36.05
	MM-R	183.66	98.79	170.86	84.63	0.42	0.67	−57.40	37.70
	MM-L	182.63	91.12	168.66	85.87	0.66	0.51	−58.50	32.70
	MM mean	183.14	91.99	169.76	81.07	0.51	0.61	−57.75	33.50
	SCM-R	13.95	6.39	13.05	6.97	0.74	0.46	−3.80	1.80
	SCM-L	14.44	9.71	13.24	9.18	0.82	0.41	−4.50	2.40
	SCM mean	14.20	7.51	13.15	7.50	0.88	0.38	−3.85	2.20
	DA-R	25.39	9.78	22.08	10.06	1.48	0.14	−8.90	1.30
	DA-L	27.84	15.64	22.83	15.00	2.20	0.03 * ES = 0.32	−9.40	−0.80
	DA mean	26.62	11.10	22.45	10.94	1.99	0.04 * ES = 0.29	−9.05	−0.25
Maximum Mouth Opening	TA-R	5.53	3.31	5.33	2.79	0.26	0.79	−1.40	1.20
	TA-L	5.20	2.85	4.49	1.73	0.76	0.45	−1.50	0.70
	TA mean	5.36	2.73	4.91	2.02	0.52	0.61	−1.50	0.90
	MM-R	7.81	6.18	7.47	4.89	−0.12	0.90	−1.50	1.60
	MM-L	7.63	5.33	7.30	4.81	0.03	0.97	−1.40	1.50
	MM mean	7.72	5.29	7.38	4.35	−0.08	0.94	−1.40	1.60
	SCM-R	6.91	3.74	7.05	3.50	−0.24	0.81	−1.60	1.90
	SCM-L	6.17	3.14	6.80	3.32	−0.82	0.41	−0.90	2.10
	SCM mean	6.54	3.34	6.93	3.25	−0.43	0.67	−1.15	1.95
	DA-R	68.11	38.00	74.00	42.48	−0.40	0.69	−15.50	24.20
	DA-L	69.70	43.82	74.89	43.98	−0.60	0.55	−16.60	27.80
	DA mean	68.90	39.90	74.45	41.36	−0.54	0.59	−16.25	27.00

TA—the anterior part of the temporalis muscle; MM—the superficial part of the masseter muscle; SCM—the middle part of the sternocleidomastoid muscle; DA—the anterior belly of the digastric muscle; R—right site; L—left site; ES—Effect Size; * Significant difference.

**Table 3:** Results of the comparison of bioelectrical patterns of selected muscles of the masticatory and cervical spine muscles in women.

		Open Eye		Closed Eye					
		Mean	SD	Mean	SD	Z	P	CI 95%	
Rest	AsI-TA	0.00	0.25	0.01	0.21	0.00	1.00	−0.11	0.11
	AsI-MM	−0.02	0.16	−0.02	0.25	−0.34	0.74	−0.08	0.12
	AsI-SCM	−0.02	0.13	0.01	0.17	−1.05	0.29	−0.03	0.11
	AsI-DA	0.01	0.09	0.04	0.11	−1.40	0.16	−0.01	0.08
	AcI-R	0.00	0.34	0.04	0.33	−0.56	0.57	−0.13	0.22
	AcI-L	0.01	0.31	0.07	0.37	−0.40	0.69	−0.12	0.24
	AcI mean	0.00	0.31	0.07	0.34	−0.78	0.44	−0.10	0.23
Clenching in The Intercuspal Position	AsI-TA	0.05	0.20	0.02	0.18	0.50	0.61	−0.11	0.06
	AsI-MM	0.03	0.14	0.05	0.15	−0.22	0.82	−0.05	0.07
	AsI-SCM	0.01	0.18	0.02	0.21	−0.19	0.85	−0.08	0.10
	AsI-DA	0.00	0.21	0.03	0.22	−0.80	0.42	−0.06	0.15
	AcI-R	0.00	0.23	−0.03	0.23	0.80	0.42	−0.14	0.07
	AcI-L	0.02	0.28	−0.05	0.25	1.07	0.29	−0.19	0.06
	AcI mean	0.01	0.23	−0.04	0.21	0.85	0.39	−0.15	0.06
Clenching on Dental Cotton Rollers	AsI-TA	0.00	0.19	0.02	0.22	−0.30	0.76	−0.08	0.10
	AsI-MM	−0.01	0.13	0.00	0.15	−0.19	0.85	−0.05	0.08
	AsI-SCM	0.02	0.18	0.04	0.20	−0.15	0.88	−0.08	0.12
	AsI-DA	−0.03	0.20	0.00	0.22	−0.56	0.58	−0.08	0.14
	AcI-R	0.12	0.23	0.11	0.21	0.53	0.60	−0.13	0.10
	AcI-L	0.14	0.21	0.12	0.20	0.54	0.59	−0.12	0.09
	AcI mean	0.13	0.19	0.11	0.18	0.34	0.73	−0.12	0.08
Maximum Mouth Opening	AsI-TA	0.03	0.17	0.06	0.17	−0.49	0.62	−0.06	0.11
	AsI-MM	0.00	0.16	0.01	0.17	−0.62	0.54	−0.05	0.09
	AsI-SCM	0.05	0.13	0.02	0.17	0.59	0.55	−0.10	0.05
	AsI-DA	0.00	0.11	0.00	0.13	0.09	0.93	−0.06	0.05
	AcI-R	0.13	0.28	0.14	0.21	0.06	0.95	−0.12	0.11
	AcI-L	0.16	0.22	0.19	0.19	−0.36	0.72	−0.07	0.10
	AcI mean	0.14	0.24	0.17	0.17	−0.11	0.91	−0.09	0.10

AcI—Activity index; AsI—Asymmetry Index; TA—the anterior part of the temporalis muscle; MM—the superficial part of the masseter muscle; SCM—the middle part of the sternocleidomastoid muscle; DA—the anterior belly of the digastric muscle; R—right site; L—left site.

**Table 4:** Results of comparison of bioelectrical activity of selected muscles of the masticatory and cervical spine muscles in men.

		Open Eye		Closed Eye					
		Mean	SD	Mean	SD	Z	P	CI 95%	
Rest	TA-R	2.63	2.01	2.30	1.98	0.43	0.67	−1.66	0.37
	TA-L	2.21	1.72	1.74	1.16	0.41	0.68	−1.09	0.36
	TA mean	2.42	1.63	2.02	1.42	0.65	0.52	−1.23	0.33
	MM-R	3.19	2.57	2.74	1.87	0.16	0.87	−1.31	0.90
	MM-L	2.54	1.45	2.39	1.49	0.46	0.65	−1.02	0.75
	MM mean	2.86	1.87	2.57	1.54	0.36	0.72	−1.23	0.76
	SCM-R	1.13	0.39	1.19	0.49	−0.21	0.84	−0.22	0.27
	SCM-L	1.30	0.33	1.25	0.37	0.27	0.79	−0.30	0.21
	SCM mean	1.22	0.33	1.22	0.40	−0.02	0.99	−0.25	0.25
	DA-R	1.48	0.56	1.40	0.53	0.25	0.80	−0.45	0.31
	DA-L	1.47	0.54	1.39	0.49	0.25	0.80	−0.43	0.31
	DA mean	1.47	0.54	1.39	0.51	0.18	0.85	−0.42	0.29
Clenching in The Intercuspal Position	TA-R	156.25	65.99	143.74	56.16	0.90	0.37	−57.70	34.90
	TA-L	142.98	72.78	132.44	63.16	0.55	0.58	−60.70	34.90
	TA mean	149.62	66.13	138.09	54.70	0.65	0.52	−0.42	0.29
	MM-R	171.81	109.05	144.59	102.25	0.87	0.38	−87.90	27.40
	MM-L	150.90	87.45	133.01	77.24	0.38	0.70	−74.60	40.10
	MM mean	161.35	93.27	138.80	83.42	0.68	0.50	−68.40	24.15
	SCM-R	11.17	7.40	9.38	5.54	0.82	0.41	−4.40	1.80
	SCM-L	14.24	14.68	11.10	10.29	0.84	0.40	−5.90	2.90
	SCM mean	12.71	10.74	10.24	7.67	1.03	0.30	−5.60	2.05
	DA-R	19.88	14.98	17.28	14.92	0.60	0.55	−9.30	4.30
	DA-L	18.76	12.08	16.76	12.40	0.71	0.48	−9.10	4.60
	DA mean	19.32	13.32	17.02	13.41	0.78	0.43	−7.35	3.40
Clenching on Dental Cotton Rollers	TA-R	152.24	73.08	148.84	62.24	−0.05	0.96	−51.20	50.60
	TA-L	140.56	57.72	140.38	57.92	−0.17	0.86	−40.00	42.90
	TA mean	146.40	62.88	144.61	57.96	0.14	0.89	−41.80	44.90
	MM-R	193.12	73.16	187.03	89.92	0.63	0.53	−61.10	35.60
	MM-L	177.98	66.32	170.36	64.20	0.36	0.72	−48.70	37.10
	MM mean	185.55	58.40	178.69	69.86	0.74	0.46	−56.10	33.00
	SCM-R	12.68	7.36	11.39	6.84	0.63	0.53	−5.80	3.00
	SCM-L	15.44	12.52	14.45	11.91	0.63	0.53	−5.30	3.70
	SCM mean	14.06	9.51	12.92	8.95	0.74	0.46	−5.00	3.35
	DA-R	19.19	10.33	16.96	8.47	0.53	0.59	−7.90	4.40
	DA-L	18.74	7.91	16.39	6.22	0.70	0.48	−7.90	4.30
	DA mean	18.96	8.58	16.68	6.77	0.56	0.58	−8.05	4.35
Maximum Mouth Opening	TA-R	8.47	9.81	5.82	4.83	0.33	0.74	−2.50	1.40
	TA-L	8.30	8.52	5.70	4.87	0.71	0.48	−3.60	1.80
	TA mean	8.39	8.37	5.76	4.66	0.62	0.54	−3.40	1.45
	MM-R	13.89	16.58	16.87	23.43	−0.22	0.82	−5.30	6.40
	MM-L	11.99	12.21	14.38	23.26	−0.30	0.76	−4.70	4.60
	MM mean	12.94	13.57	15.63	15.14	−0.74	0.46	−4.20	8.15
	SCM-R	13.29	10.49	12.03	11.10	0.49	0.62	−7.80	4.20
	SCM-L	16.09	18.58	13.86	15.72	0.28	0.78	−7.70	4.90
	SCM mean	14.69	13.02	12.94	11.76	0.46	0.65	−7.85	4.55
	DA-R	50.18	43.19	50.60	46.69	−0.10	0.92	−34.20	35.70
	DA-L	44.28	36.88	43.85	39.40	0.00	1.00	−26.30	22.50
	DA mean	47.23	39.89	47.22	42.92	−0.10	0.92	−29.75	26.35

TA—the anterior part of the temporalis muscle; MM—the superficial part of the masseter muscle; SCM—the middle part of the sternocleidomastoid muscle; DA—the anterior belly of the digastric muscle; R—right site; L—left site; ES—Effect Size;.

**Table 5:** Results of comparison of bioelectrical patterns of selected muscles of the masticatory and cervical spine muscles in men.

		Open Eye		Closed Eye					
		Mean	SD	Mean	SD	Z	P	CI 95%	
Rest	AsI-TA	0.07	0.26	0.08	0.23	0.02	0.99	−0.15	0.17
	AsI-MM	0.05	0.22	0.05	0.20	0.11	0.91	−0.16	0.13
	AsI-SCM	−0.08	0.11	−0.04	0.12	−0.90	0.37	−0.05	0.12
	AsI-DA	0.00	0.09	0.00	0.05	0.55	0.58	−0.06	0.04
	AcI-R	0.08	0.36	0.11	0.37	−0.40	0.69	−0.25	0.29
	AcI-L	0.12	0.28	0.15	0.28	0.02	0.99	−0.17	0.24
	AcI mean	0.09	0.31	0.13	0.32	−0.52	0.60	−0.17	0.26
Clenching in The Intercuspal Position	AsI-TA	0.07	0.14	0.06	0.16	0.43	0.67	−0.13	0.09
	AsI-MM	0.08	0.24	0.04	0.27	0.81	0.42	−0.19	0.09
	AsI-SCM	−0.05	0.22	−0.01	0.27	−0.36	0.72	−0.15	0.18
	AsI-DA	0.01	0.16	0.00	0.19	0.00	1.00	−0.12	0.12
	AcI-R	−0.01	0.28	−0.07	0.31	0.46	0.65	−0.27	0.14
	AcI-L	−0.02	0.26	−0.07	0.27	0.52	0.60	−0.19	0.11
	AcI mean	0.00	0.25	−0.06	0.25	0.59	0.56	−0.21	0.13
Clenching on Dental Cotton Rollers	AsI-TA	0.02	0.11	0.02	0.11	−0.02	0.99	−0.08	0.09
	AsI-MM	0.04	0.19	0.03	0.17	0.11	0.91	−0.12	0.11
	AsI-SCM	−0.07	0.21	−0.09	0.22	0.40	0.69	−0.15	0.13
	AsI-DA	0.00	0.16	0.00	0.18	0.02	0.98	−0.11	0.16
	AcI-R	0.14	0.26	0.12	0.24	0.46	0.65	−0.21	0.14
	AcI-L	0.13	0.19	0.11	0.20	0.68	0.50	−0.13	0.07
	AcI mean	0.14	0.20	0.12	0.20	0.46	0.65	−0.15	0.09
Maximum Mouth Opening	AsI-TA	0.01	0.22	0.04	0.18	0.17	0.86	−0.12	0.13
	AsI-MM	0.02	0.21	0.04	0.39	−0.11	0.91	−0.16	0.17
	AsI-SCM	−0.04	0.24	−0.03	0.24	−0.11	0.91	−0.16	0.16
	AsI-DA	0.05	0.10	0.05	0.09	0.10	0.92	−0.09	0.08
	AcI-R	0.17	0.31	0.26	0.37	−0.87	0.38	−0.17	0.31
	AcI-L	0.16	0.27	0.29	0.29	−1.15	0.25	−0.09	0.30
	AcI mean	0.17	0.27	0.32	0.35	−1.12	0.26	−0.11	0.36

AcI—Activity index; AsI—Asymmetry Index; TA—the anterior part of the temporalis muscle; MM—the superficial part of the masseter muscle; SCM—the middle part of the sternocleidomastoid muscle; DA—the anterior belly of the digastric muscle; R—right site; L—left site.

## Data Availability

The datasets generated during and/or analyzed during the current study are available from the corresponding author on reasonable request.
